# Torsade de Pointes Induced by Hypokalemia from Imipenem and Piperacillin

**DOI:** 10.1155/2017/4565182

**Published:** 2017-05-30

**Authors:** Varun Kumar, Sandeep Khosla, Monica Stancu

**Affiliations:** Mount Sinai Hospital, Department of Cardiology, Suite L629, 1500 S. California Ave, Chicago, IL 60608, USA

## Abstract

Imipenem-cilastatin and piperacillin-tazobactam are two antibiotics with broad antimicrobial coverage. Besides the many well established adverse effects of these drugs, there have been few case reports of hypokalemia. Here we present an interesting case of resistant hypokalemia caused by these drugs leading to Torsades de Pointes which has never been reported in the past. Hypokalemia resolved with discontinuation of piperacillin.

## 1. Introduction

Torsades de Pointes (TdP) is one of the life threatening arrhythmias. It is associated with prolonged QT interval. Electrolyte abnormalities are one of the common causes of prolongation of QT interval. Here we present a rare instance of imipenem-cilastatin (IC) and piperacillin-tazobactam (PT) causing hypokalemia leading to this potentially lethal ventricular tachycardia (VT).

## 2. Case

**Figure 1:**
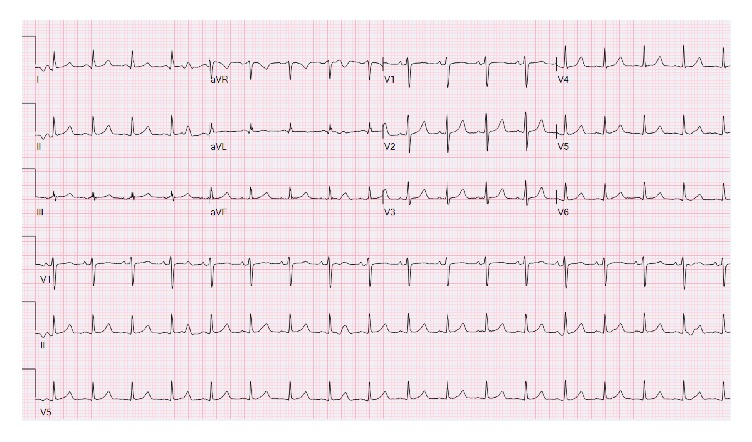
QTc 441 (Day 1).

A 39-year-old female presented as a transfer from outside hospital for management of peritonitis and peritoneal abscess following colon perforation which occurred as a complication from oophorectomy and ventral hernia repair. Patient underwent exploratory laparotomy and diverting colostomy and a drain was placed for drainage of peritoneal abscess. Electrocardiogram (ECG) (Figure 1) at admission demonstrated normal QT interval and serum potassium was 5.2 mEq/L. No structural abnormalities were noted on echocardiogram.

**Figure 2:**
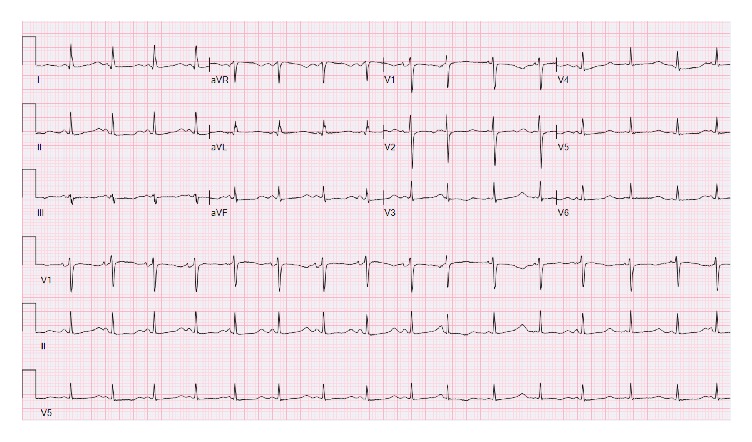
QTc 632 (Day 8).

After 6 days of treatment with intravenous IC, she developed hypokalemia of 3.1 mEq/L, metabolic alkalosis, and prolongation of QT interval (QTc 533 ms). The rest of the electrolytes including magnesium and serum calcium were normal. Potassium was replaced and the antibiotic was switched to PT based on cultures after 2 days. Hypokalemia worsened to 2.9 mEq/L and continued to remain low despite replacement of potassium of 80–120 mEq/day. QTc further increased to 632 ms (Figure 2). No QT prolonging drug was administered in the previous 48 hours (see the following list). 

List of medications administered before the episode of Torsades de Pointes (TdP):

- Acetaminophen
- Amlodipine besylate
- Calcium gluconate
- Chlorhexidine gluconate (last administered 5 days before episode of TdP)
- Dexmedetomidine hydrochloride (last administered 5 days before episode of TdP)
- Divalproex sodium
- Docusate sodium with senna
- Enoxaparin injection
- Fentanyl citrate in 0.9% NaCl (last administered 5 days before episode of TdP)
- Furosemide injection (last administered 5 days before episode of TdP)
- Hydromorphone hydrochloride injection (last administered 5 days before episode of TdP)
- Imipenem-cilastatin (last administered 2 days before episode of TdP)
- Lisinopril
- Lorazepam injection (last administered 9 days before episode of TdP)
- Metoclopramide hydrochloride (last administered 6 days before episode of TdP)
- Micafungin sodium (last administered 13 days before episode of TdP)
- Midazolam hydrochloride (last administered 5 days before episode of TdP)
- Morphine sulfate (last administered 10 days before episode of TdP)
- Olanzapine injection (last administered 4 days before episode of TdP)
- Pantoprazole sodium (last administered 4 days before episode of TdP)
- Piperacillin-tazobactam
- Potassium chloride
- Potassium phosphate
- Sertraline hydrochloride (last administered 3 days before episode of TdP)
- Vancomycin hydrochloride (last administered 5 days before episode of TdP).

**Figure 3:**
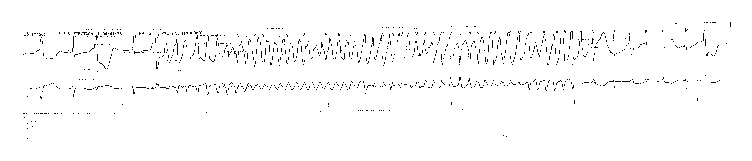
Torsades de Pointes (Day 8).

**Figure 4:**
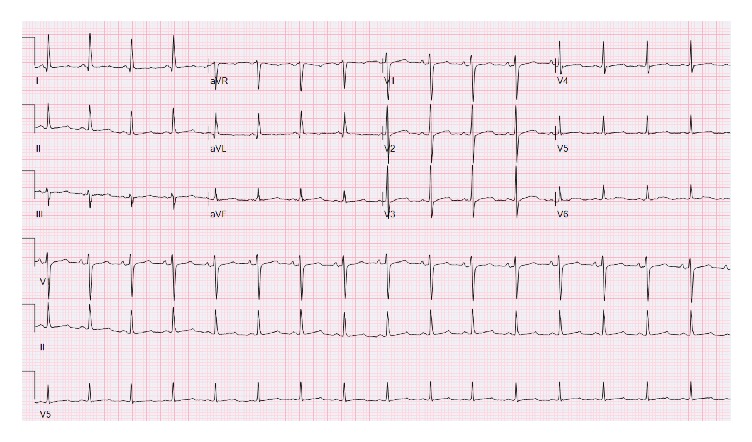
QTc 487 (Day 17).

Three episodes of self-terminating TdP were noted on telemetry, with approximately ten seconds being the longest duration (Figure 3). During these episodes, the patient experienced palpitations, dyspnea, and lightheadedness. Multiple episodes of nonsustained VT were also noted on telemetry. Intravenous magnesium was administered and potassium replenishment continued. Transtubular potassium gradient was calculated to be 6.5 indicating renal loss of potassium. Renin and aldosterone levels were normal. Repeat cultures demonstrated* Enterobacter cloacae* which was resistant to PT in addition to* Proteus mirabilis* and* Enterococcus faecalis*. PT was switched back to IC as per recommendations by infectious disease specialist. Hypokalemia now responded to potassium replacement and improved to 4 mEq/L from 3.3 mEq/L and remained in normal range. QTc improved to 487 (Figure 4) and patient had no more events on telemetry. It has to be noted that during her entire hospitalization she did not develop diarrhea or vomiting to suspect gastrointestinal loss of potassium or acid.

## 3. Discussion

QT interval which is the time interval between beginning of Q wave and end of T wave represents electrical depolarization and repolarization of ventricles. The corrected QT interval (QTc) estimates the QT interval at a heart rate of 60 beats per minute which enables comparison of QT intervals at different heart rates. QTc is calculated using Bazett's formula (QTc = QT/√RR interval). QTc of less than 430 ms in males and less than 450 ms in females are considered to be normal [1]. Prolongation of QT interval may be noted when there is a delay in myocardial repolarization secondary to ionic currents from electrolyte abnormalities. Phase 3 of myocardial repolarization is predominantly mediated through delayed outward rectifier potassium currents (*I*_Kr_ and *I*_Ks_) which are in turn dependent on extracellular potassium concentration [2, 3]. In case of hypokalemia, there is decreased expression of these channels resulting in prolongation of repolarization [4]. This can cause early afterdepolarizations (EADs) from inward depolarizing currents through T-type calcium channels or sodium channels which appear as U waves on ECG. When these EADs reach threshold potential, they trigger premature action potential. Additionally, there could be heterogeneity of repolarization in myocardial cells especially in Purkinje cells and M cells in mid ventricular myocardium. EADs along with this dispersion of repolarization can initiate reentrant mechanism leading to ventricular tachyarrhythmias such as TdP [5, 6].

TdP refers to polymorphic ventricular tachycardia where QRS complexes appear to twist around an isoelectric line in a sinusoidal pattern [7]. This potentially life threatening arrhythmia is usually short lived and self-terminating. However, when it occurs in quick successions, it can progress to ventricular fibrillation and sudden cardiac death [8, 9]. Some of the risk factors for prolongation of QT interval leading to TdP are as follows:

1. Congenital prolongation of QT interval (LQTS): Jervell and Lange-Nielsen syndrome and Romano Ward syndrome
2. Electrolyte abnormalities: hypokalemia [10], hypomagnesemia [11], and hypocalcemia [12–14]
3. Structural heart disease: congestive heart failure and early phase of myocardial ischemia [14]
4. Bradycardia [15]
5. Drugs:
  - Antiarrhythmic drugs: Class 1A (quinidine, procainamide, and disopyramide), Class 1C (encainide, flecainide), and Class 3 (sotalol, dofetilide)
  - Psychotropic drugs: haloperidol, methadone, and lithium
  - Antibiotics: erythromycin and azithromycin
  - Anthracycline chemotherapeutic agents: doxorubicin and daunomycin
  - Antiemetic drugs: ondansetron.

In the patient above, hypokalemia was the cause for prolonged QT interval with episodes of TdP. IC and PT are antibacterial agents which are structurally similar to penicillin and both have *β*-lactam ring. They are eliminated primarily through kidneys. Hypokalemia and metabolic alkalosis are one of the rare adverse effects of these drugs. The two drugs behave as nonabsorbable anions in the renal tubules leading to electronegativity across the tubule. This results in increased delivery of sodium to distal nephron which in turn stimulates aldosterone causing absorption of sodium and secretion of potassium and hydrogen [16–18]. In the above patient, serum sodium and carbon dioxide levels increased to 151 mEq/L and 33 mEq/L, respectively, and potassium levels dropped to 2.9 mEq/L. Hypokalemia was relatively resistant to potassium replacements. Once the patient was switched from PT to IC based on culture and sensitivity, hypokalemia responded to potassium replacement and hypernatremia resolved.

It is unclear if PT has higher tendency to cause hypokalemia than IC. There have been few case reports of PT causing hypokalemia [18–20]. Though there have been reports of the same with structurally similar meropenem [17], there have been no such reports with IC besides causing alkalosis [17, 21]. In our patient, mild hypokalemia and alkalosis developed first with IC. On switching to PT, hypokalemia worsened and remained resistant to replenishment along with development of hypernatremia. The battle with hypokalemia resolved once PT was switched back to IC. Patient remained alkalotic at the time of discharge to long term care facility (LTC) on IC.

Though the patient had no prior history of syncopal episodes, family history of sudden cardiac death, or LQTS, there is a possibility that the patient may have had undiagnosed LQTS and hypokalemia may have unmasked this, as the QT interval was still mildly prolonged at the time of discharge even after the correction of hypokalemia. Our facility did not have the capability for genetic testing and patient was given a referral for outpatient testing. However, patient was lost to follow-up after she was discharged from LTC.

## 4. Conclusion

Though electrolyte abnormalities causing prolonged QT interval and potentially fatal TdP are well established, physicians while treating patients with antibiotics such as IC and PT should be aware that they can cause difficult-to-treat hypokalemia with cardiac effects. The lacking of the full characterization of these drugs at the cellular model could have an enormous potential for in vitro studies.
